# The challenge of the differential diagnosis between brown tumors and metastases in parathyroid carcinoma: a case report

**DOI:** 10.3389/fendo.2024.1414896

**Published:** 2024-10-18

**Authors:** Elisa Dinoi, Alessandro Prete, Chiara Sardella, Laura Pierotti, Simone Della Valentina, Anna Dal Lago, Simona Borsari, Elena Pardi, Maria Adelaide Caligo, Angela Michelucci, Liborio Torregrossa, Piercarlo Rossi, Filomena Cetani

**Affiliations:** ^1^ Department of Clinical and Experimental Medicine, University of Pisa, Pisa, Italy; ^2^ Unit of Endocrinology, University Hospital of Pisa, Pisa, Italy; ^3^ Laboratory of Molecular Genetics, University Hospital of Pisa, Pisa, Italy; ^4^ Department of Surgical, Medical, Molecular Pathology and Clinical Area, University Hospital of Pisa, Pisa, Italy; ^5^ Radiology, Department of Translational Research on New Technologies in Medicine and Surgery, University of Pisa, Pisa, Italy

**Keywords:** hyperparathyroidism, brown tumors, bone metastases, parathyroid carcinoma, hungry bone

## Abstract

**Background:**

Brown tumors are rare bone manifestations of primary hyperparathyroidism (PHPT) that may occur at different sites either as single or multiple lesions and they can easily be mistaken for malignant lesions. Neither bone site nor morphological or functional imaging are useful to drive the differential diagnosis and biopsy is often the only conclusive procedure.

**Case description:**

We report the case of a 53 years-old man referred to our outpatient clinic for severe symptomatic PHPT complicated by nephrolithiasis and osteoporosis. Neck ultrasound and computed tomography (CT) scan showed a large irregular lesion posterior to the lower pole of the right thyroid lobe consistent with an enlarged parathyroid gland. Moreover, two bone lytic lesions were described at the left scapula and the contiguous 7^th^ rib that showed an increased uptake at total bone scintigraphy. Given the clinical and biochemical picture, the features of the parathyroid lesion and the presence of bone lytic lesions, the suspicion of metastatic parathyroid carcinoma (PC) was raised. However, a CT-guided biopsy performed on the left scapula revealed a brown tumor. The patient underwent en-bloc resection of the right inferior parathyroid grand with the ipsilateral thyroid gland lobe. Histopathology confirmed the diagnosis of PC. Post-surgical biochemical evaluations showed that the patient was cured. A repeated total body CT scan revealed an osteoblastic appearance of the bone lesions ascribed to the partial regression of the brown tumors following surgery.

**Conclusions:**

The implication of a diagnosis of brown tumor or bone metastasis is widely different; in fact, the first tends to regress with the surgical treatment of PHPT, whereas the latter has limited cure option and negatively affects the prognosis of patients. Therefore, although brown tumors are extremely rarer than in the past, they must always be taken into consideration in the presence of bone lesions, even in cases of high suspicion of malignancy, to avoid unnecessary and harmful surgical interventions.

## Introduction

Primary hyperparathyroidism (PHPT) is the third more common endocrine disorder, and it is characterized by hypercalcemia and elevated or inappropriately normal parathyroid hormone (PTH) levels, due to uncontrolled secretion by one or multiple parathyroid glands (1). Since the advent of multichannel autoanalyzer, PHPT has become mostly an asymptomatic disorder characterized by mild hypercalcemia (2). Severe parathyroid bone disease is rarely seen in the United States and Western Europe and a trend towards a milder form of disease presentation has also been reported in developing countries (3, 4). However, osteitis fibrosa cystica (OFC), the classical bone disease of PHPT, still occurs all over the world (5, 6). The pathology of OFC is characterized by excessive osteoclast resorption and destruction of the cortical compartment with the formation of fibrous cysts; vascularized fibrous tissue and osteoclast-giant cells may substitute the bone marrow (7). Brown tumors represent a localized form of OFC. The term derives from the accumulation of blood pigment which gives the reddish-brown appearance at histology (7). They commonly arise in the pelvis, ribs, clavicles and extremities (8), though atypical locations have been reported (9–11). On imaging, brown tumors usually appear as lytic lesions; therefore, being rare, they are often not included in the differential diagnosis and misdiagnosed as other bone diseases (12, 13).

In parathyroid cancer (PC), a rare cause of PHPT (<1% of all cases), skeletal manifestations affect up to 90% of patients (14). When bone lytic lesions are detected in patients with a high suspicion of PC, the differential diagnosis between brown tumors and bone metastatic lesions may be challenging (15).

We present the case of a 53-years-old man suffering from severe symptomatic PHPT.

During a computed tomography (CT) scan, two bony lytic lesions were discovered, prompting concerns of metastatic PC. However, biopsy of one of the skeletal lesions confirmed the presence of a brown tumor. Following successful surgery, the bone lesions gradually resolved.

This case emphasizes how such rare and multiple benign lesions represent a real challenge for the clinician in the differential diagnosis with metastatic lesions. Physicians should be aware that brown tumors exhibit hypermetabolic activity and appear positive on functional imaging, Furthermore, this case also documents that successful surgery leads to the resolution of bone lesions associated with severe PHPT.

## Case description

**Figure 1:**
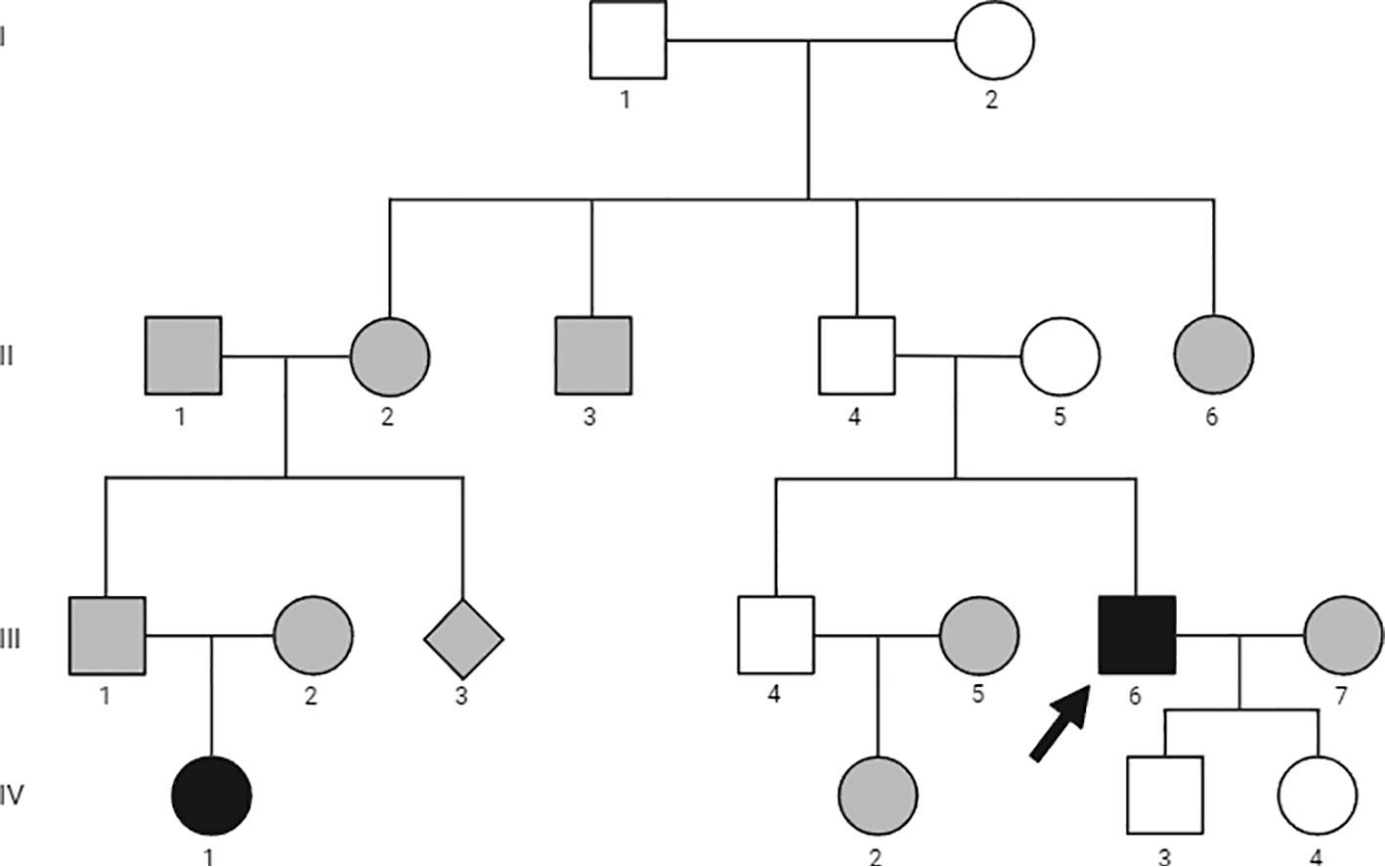
Family pedigree. Family members are indicated by generation and individual numbers, respectively. Circles represent women, squares men, filled symbols affected family members, empty symbols unaffected members, gray symbols not evaluated. The arrow identifies the proband.

A 53-yr-old man (**Figure 1**, III-6) was referred to our outpatient clinic in February 2021 for further evaluation of PHPT which was incidentally discovered during investigation for knee pain. He also reported marked fatigue, which had developed a year earlier and was initially attributed to suspected algodystrophy, for which he had been previously treated with clodronate. The diagnosis of PHPT was established on severe hypercalcemia (14.9 mg/dL; normal range 8.4-10.4) and markedly high plasma PTH levels (581 pg/mL; normal range 6.5-36.8).

His past medical history was remarkable for the presence of symptomatic bilateral nephrolithiasis since the age of 47 years. The patient was also affected by arterial hypertension and gastroesophageal reflux. His family history was positive for PHPT in the daughter of a paternal cousin (**Figure 1**, IV-1). She had already undergone parathyroidectomy in another institution with the resection of a single chief cell parathyroid adenoma. She was not affected by other endocrine disorders and was not available for further evaluation. There was no family history of parathyroid disease or other endocrinopathy, urolithiasis, or peptic ulcer disease in other family members.

At the time of our evaluation, the patient was receiving therapy consisting with valsartan 40 mg daily for arterial hypertension and omeprazole 20 mg daily for gastroesophageal reflux. On physical examination, the patient was in fair general condition. His pulse rate was 75 beats/min and blood pressure 120/70 mmHg. No masses were palpable in the cervical region.

Laboratory investigations confirmed the diagnosis of PHPT with severe hypercalcemia and markedly elevated PTH levels (**Table 1**). Twenty-four-hour urinary calcium and bone turnover markers were also markedly increased (**Table 1**). Prolactin, GH, IGF-I, TSH, ACTH, cortisol, insulin, gastrin, and glucose concentrations were within the normal range (data not shown). Renal ultrasound and CT scan detected bilateral nephrolithiasis. Bone mineral density (BMD) measured by dual-energy X-ray absorptiometry (DXA) showed a marked reduction in bone mass at all sites: lumbar BMD 0,651 g/cm^2^, T-score -4.0; total hip BMD 0,549 g/cm^2^, T-score -3.2; femoral neck BMD 0,424 g/cm^2^, T-score -3.7; 1/3 distal radius BMD 0,405 g/cm^2^, T-score -7.8. There were no signs of vertebral fracture at vertebral fracture assessment (VFA).

**Table 1:** Biochemical parameters at baseline and after surgery.

Before Surgery				After Surgery				
Analyte	Baseline	1 week after ZOL	1 week after CINA	1 month	6 months	12 months	22 months	Reference range
**Total calcium (mg/dL)**	13.6	11	9.1	8.6	9	9.6	9.3	8.6-10.2
**Ionized calcium (mmol/L)**	2.11			1.11	1.18	1.27	1.17	1.13-1.32
**Phosphate (mg/dL)**	1.5	0.9	1.2	2.1	4	2.7	3.2	2.5-4.5
**Magnesium (mg/dL)**	2.0			NA	NA	2.0	2.2	1.7-2.5
**Creatinine (mg/dL)**	0.92			NA	NA	0.89	0.87	0.7-1.2
**eGFR**	94.6					96.9	97.2	
**PTH (pg/mL)**	352			21	20	28	28	8-40
**25(OH)D (ng/mL)**	23			17.6	31	22.5	20.6	20-100
**BSAP (mcg/L)**	150			58	27	23	17	4.7-27.1
**Osteocalcin (mcg/L)**	192			77	NA	13.1	5.7	6.8-34
**S-CTX (mcg/L)**	NA			0.062	<0.033	<0.033	0.067	0.038- 0.724
**24h urinary calcium (mg/24h)**	420			NA	NA	184	NA	100-320

ZOL, Zoledronic Acid; CINA, Cinacalcet 30 mg BID, eGFR, estimated glomerular filtration rate; 25(OH)D, 25-hydroxyvitamin D; BSAP, bone specific alkaline phosphatase; S-CTX, serum carboxy-terminal collagen crosslinks; NA, not available.

**Figure 2:**
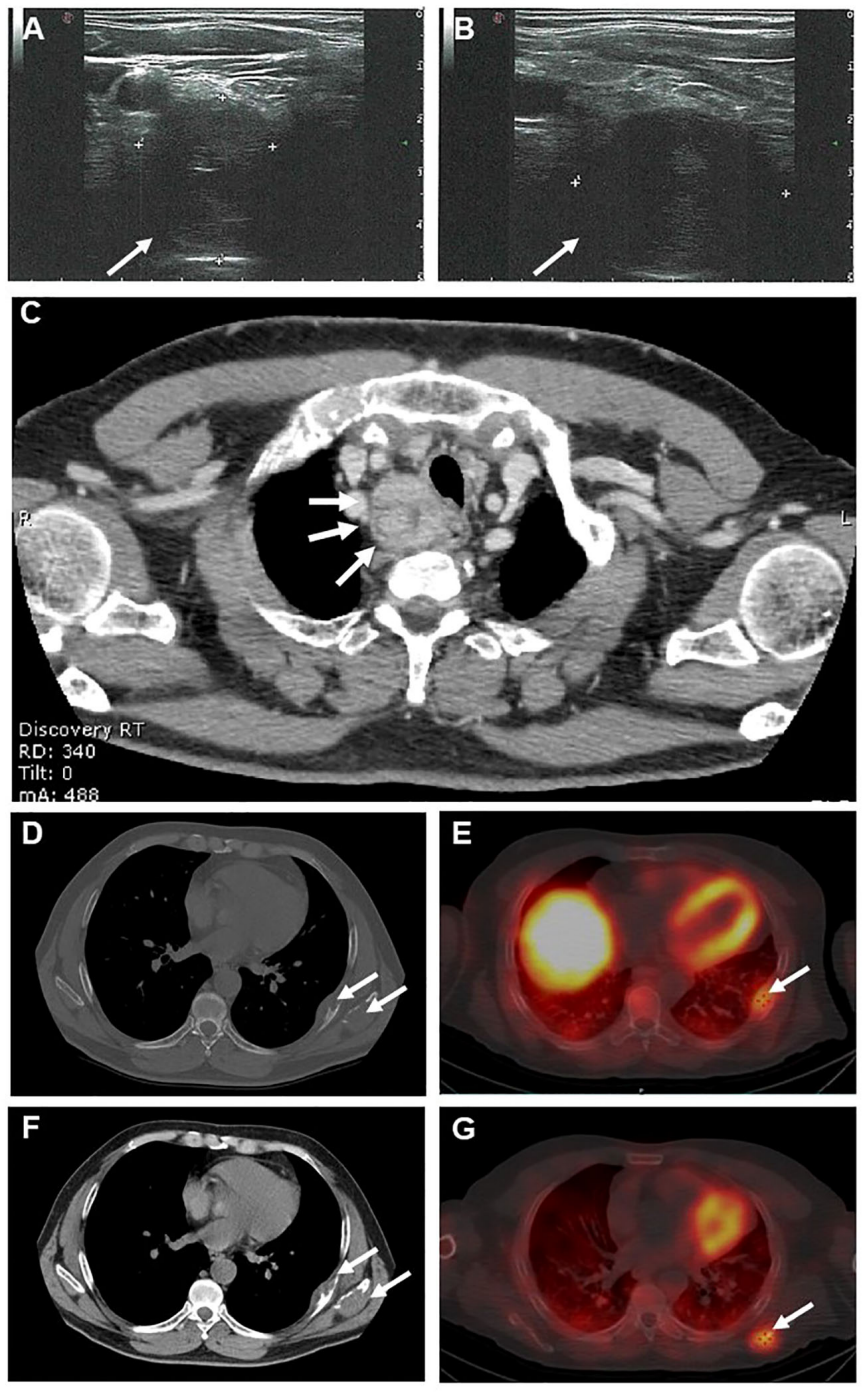
Neck ultrasound showed a 3-cm solid non-homogeneous hypoechoic nodular lesion with irregular borders, posterior to the lower pole of the right thyroid lobe consistent with an enlarged parathyroid gland **(A, B)**. Axial contrast-enhanced neck computed tomography (CT) **(C)** showing a 3-cm solid heterogeneous lesion with a central area of hypodensity and irregular borders located below the right thyroid lobe (arrows). Axial section basal **(D)** and contrast-enhanced **(F)** CT scans showing two osteolytic lesions at the inferior border of the left scapula(arrow) and in the 7th rib (arrow). Axial section ^99m^Tc-sestamibi SPECT-CT scan showing uptake at the 7^th^ rib (**E**, arrow) and the left scapula (**G**, arrow).

Neck ultrasound showed a 3-cm solid non-homogeneous hypoechoic nodular lesion with irregular borders, posterior to the lower pole of the right thyroid lobe consistent with an enlarged parathyroid gland (**Figures 2A, B**). Total body contrast-enhanced computed tomography (CT) scan confirmed the presence in the neck of a firm lesion of heterogeneous structure with a central area of hypodensity and irregular borders located in the superior mediastinum below the right thyroid lobe, with no signs of local infiltrations (**Figure 2C**). Moreover, CT scan revealed two osteolytic lesions in the inferior border of the left scapula and the contiguous 7th rib interpreted by the radiologist as metastatic lesions and no other pathological findings (**Figures 2D–F**). ^99m^Tc-sestamibi dual-phase planar scintigraphy scan and single-photon emission computed tomography (SPECT-CT) scan showed late uptake at the right neck and at the sites of the skeletal lesions (**Figure 2G**). Total body bone scintigraphy (^99^mTc-HDP 791 MBq) revealed high uptake of both bone lesions.

**Figure 3:**
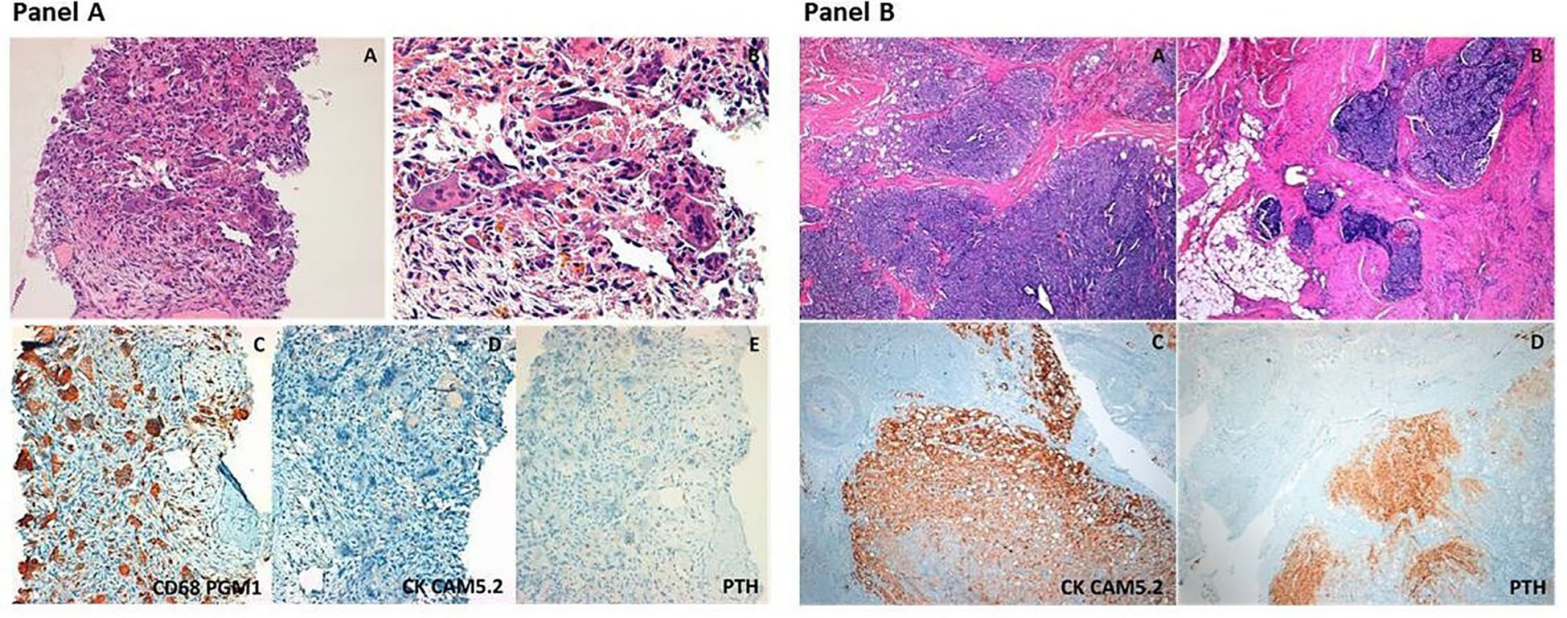
Panel **(A)** Representative histopathologic images of the scapular osteolytic lesion. The so-called “brown tumor” consists of accumulation of giant cells, including osteoclasts and foreign body giant cells, around areas of hemosiderin deposition and hemorragic foci (A, B hematoxylin&eosin stain, original magnification 20× and 40x, respectively). Immunohistochemical stainings show intense positivity for CD68 (clone PGM1, that is an immunomarker of macrophages and other mononuclear phagocytes) (C), and absence of immunoreactivity for cytokeratins (CK CAM5.2) and Parathyroid Hormone (PTH), (D, E, original magnification 20×). Panel **(B)** Representative histopathologic images of the parathyroid carcinoma. The tumor shows solid growth pattern with marked fibrotic septa (A) and invasion of the surrounding soft tissues (B) (hematoxylin&eosin stain, original magnification 4×). Immunohistochemical staining highlights intense positivity for cytokeratins (CK CAM5.2) and Parathyroid Hormone (PTH), (C-D, original magnification 4×).

Given the clinical characteristics, particularly male gender, relatively young age and severe biochemical picture i.e severe hypercalcemia and markedly elevated PTH levels, along with the ultrasound and CT features of the parathyroid lesion, coupled with the discovery of two lytic bone lesions, suspicions of metastatic PC were raised (14, 16). A biopsy of the left scapular lesion was conducted. Measurement of PTH in fine-needle aspiration biopsy washing yielded undetectable levels. Histological examination revealed the presence of numerous multinucleated giant cells (immunohistochemistry: CD68 PGM1+, Cytokeratin CAM 5.2 -, PTH-) associated with interstitial hemorrhages and focal hemosiderin deposition consistent with the diagnosis of brown tumor (**Figure 3A**).

Medical therapy was initiated while awaiting surgery, starting with intravenous 4 mg zoledronic acid, which significantly reduced serum calcium concentrations from 13.6 mg/dL to 11 mg/dL. A week later, cinacalcet 30 mg BID was introduced, normalizing serum calcium to 9.1 mg/dL, a level maintained until surgery. Treatment with cholecalciferol (2000 IU daily) was also initiated. Several cross-sectional studies have suggested an inverse relationship between serum 25(OH)D and PTH levels, indicating that low vitamin D levels exacerbate PTH elevation. A recent meta-analysis of nine studies reported that vitamin D supplementation significantly reduces PTH and bone-specific alkaline phosphatase levels without significant changes in serum calcium, phosphate, and urinary calcium (17). Notably, vitamin D deficiency may be associated with a greater risk of post-operative hypocalcemia (18).

In March 2021 the patient underwent en-bloc resection of the right inferior parathyroid gland with the ipsilateral thyroid gland lobe. During neck exploration, no signs of local infiltration were observed. Intraoperative PTH levels decreased significantly from 649 pg/ml at baseline to 89 pg/ml at 5 min post excision representing an 86% decrease. Histopathological examination revealed a parathyroid neoplasm consisting of chief and clear cells with nuclear pleomorphism, solid, trabecular, and follicular growth pattern, fibrous bands, increased mitotic activity and capsular, vascular, and adjacent adipose tissue invasion consistent with the diagnosis of PC (**Figure 3B**). Ki-67 proliferation index was 2%.

Shortly after surgery, the patient developed hungry bone syndrome with numbness and tingling sensations in his fingers and toes and serum calcium was at 7.4 mg/dL. The patient was treated with calcium gluconate intravenous infusion. After discharge, he received high amounts of calcium carbonate (2.5 g) and calcitriol (1 mcg) which were progressively reduced, leading to the suspension of calcitriol after 2 months and continuation of calcium carbonate until it was discontinued after six months (**Table 1**).

A repeated whole body Tc^99m^-sestamibi scan six months after surgery showed no uptake in the neck but still high uptake at skeletal sites. Total body CT scan revealed an osteoblastic appearance of the bone lesions and no pathological lesions in the neck or elsewhere. These skeletal findings were attributed to partial regression of the brown tumors following surgery. A regular follow-up schedule with three-monthly assessments of serum calcium and PTH levels was recommended.

Based on the diagnosis of PC and a positive familial history for PHPT, genetic testing was performed. The details of the genetic analysis are outlined in the “Materials and Methods” section. Targeted next generation sequencing (NGS) analysis was carried out; however no pathological variants were identified. Additionally, multiplex ligation-dependent probe amplification (MLPA) did not reveal any gross deletions or duplications of the *CDC73*, *MEN1* and *CDKN1B* genes.

Screening of serum calcium in available first-degree relatives (**Figure 1**, II-5, III-4, IV-3 and IV-4) was in the normal range.

Biochemical data following surgery are summarized in **Table 1**. Clinically, the patient exhibited gradual improvement, experiencing regression of fatigue and joint pain.

At the most recent evaluation, 22 months after surgery the patient remained in remission, as indicated in **Table 1**. Both neck ultrasound and contrast-enhanced CT scans showed no evidence of lesions; CT scan demonstrated complete resolution of the previously bone lesions. DXA revealed an increase of BMD at the lumbar spine (+65%), femoral neck (+24%), and distal radius (+21%).

## Materials and methods

### Assays

Blood samples were obtained between 8 and 9 a.m. after an overnight fast. Serum calcium, magnesium, phosphate and creatinine were determined using standard methods.

Ionized calcium was measured by ion-selective electrode method (Nova 8 calcium analyzer, Nova Biomedical).Plasma PTH was measured by a third-generation assay (DiaSorin LIAISON 1-84 PTH chemiluminescent immunoassay) and serum 25-hydroxyvitamin D (25[OH]D) was measured by a chemiluminescent immunoassay (IDS-iSYS); Bone-specific alkaline phosphatase (BSAP) by immunoenzymatic assay (OCTEIA Ostase BAP; IDS Ltd., Boldon, Tyne & Wear, UK), serum N-MID osteocalcin (IDS Ltd., Boldon, UK), and S-CTX (Nordic Bioscience Diagnostics A/S, Herlev, Denmark) by ELISA. The reference ranges are reported in **Table 1**.

### Bone mineral density

BMD was measured by DXA using Hologic QDR-4500 (Hologic Inc., Waltham, MA, USA) at the lumbar spine in posterior-anterior projection (L1–L4), femoral sites, and 1/3 distal radius. The coefficients of variations were 1.1% at lumbar spine and 1.2% at femoral neck.

The evaluation of vertebral fractures was made by VFA using DXA images (two lateral scans of the vertebrae from T4 to L4). The accuracy of VFA had been validated in a previous study (19).

### Genetic analysis

Peripheral blood was collected and DNA extracted using the QIA symphony automatic instrument (QIAGEN,Germany).For the Next Generation Sequencing (NGS) analysis, we designed a panel using acustom-designed Agilent SureSelect QXT target sample library preparation and capture method (AgilentTechnologies, USA). The panel contains coding exons and flanking regions of 9 genes associated with hyperparathyroidism: *MEN1* (NM_130803), *CDKN1A* (NM_001220777), *CDKN1B* (NM_004064), *CDKN2B*(NM_004936), *CDKN2C* (NM_078626), *CDC73* (NM_024529), *GCM2* (NM_004752), *CASR* (NM_001178065), *AIP* (NM_003977). The library was sequenced with a NextSeq550Dx (Illumina, USA). Read alignment to reference genome (hg19), variant calling, and annotation were performed with the Agilent SureCall software (Agilent Technologies, USA). Finally, the sequencing coverage of each exon was analyzed in detail using IGV tool (Integrative Genomics Viewer, Broad Institute and the Regents of the University of California,USA).

To detect large genomic deletions or duplications of *MEN1*, *CDKN1B*, *CDC73* genes we used the multiplex ligation-dependent probe amplification (MLPA). MLPA reagents were obtained from MRC-Holland (The Netherlands): SALSA MLPA kit P244 for genes *MEN1*and *CDKN1B*, SALSA MLPA kit P466 for gene *CDC73*). About 100 ng of genomic DNA were used in the tests, according to the manufacturer’s protocol. Data analysis was performed using Coffalyser software (MRC-Holland, The Netherlands).

## Discussion

Brown tumors, representing the terminal manifestation of the bone remodeling process in PHPT, have been reported in less than 5% of cases in Western Countries (3). While the true prevalence of brown tumors in patients with PC remains unknown, bone involvement is common in these individuals due to elevated levels of PTH.

The molecular pathogenesis of brown tumors is not fully elucidated. Of note, a very few studies have reported somatic mutations of the *KRAS* gene in brown tumors suggesting that might play a role in their development (20, 21). To date no role of *KRAS* gene has been demonstrated in the pathogenesis of metastatic and non-metastatic PC.

The literature indicates that presenting symptoms of PC-related bony lesions often include bone pain and swelling (22–24) or pathologic fractures (25–27). These symptoms are frequently misinterpreted as other bone pathologies, such as giant cell tumor (22), multiple myeloma (25) or bone metastases (23, 28, 29). In some cases, misdiagnosis can lead to drastic measures, such as leg amputation, as was seen in one reported case where bone malignancy was suspected (30).

When dealing with a known diagnosis of severe PHPT the challenge often lies in distinguishing between brown tumors and PC metastases. This dilemma was illustrated in a case reported by Russo and colleagues involving a 42-years old female patient, who had previously already undergone parathyroidectomy for PC but presented with multiple bone lesions showing avid uptake on 18F-FDG PET (31). Similarly, in our patient, the combination of the severe clinical and biochemical manifestation along with radiological features of the neck lesion strongly suggested PC. Upon detection of two bone lesions on CT, there was high suspicion of their metastatic nature. The implication of these two diagnoses is significantly different: brown tumors tend to regress following surgical treatment of PHPT, whereas bone metastases offer limited treatment option and adversely affect patient’s prognosis. Therefore, caution must be exercised in interpreting the nature of bone lesions to ensure appropriate management.

While there are only a few case reports documenting patients with PC presenting with brown tumors, up to 30% of patients with PC are diagnosed with metastases, commonly involving the lungs and bone (32). Specifically, Talat and colleagues reported metastases in 145 of 1036 cases of PC, accounting for 13% of cases. Among these metastatic cases, 57% of patients had lung metastases, 11% bone metastases and 4% had both lung and bone metastases (33).

The site of bone involvement lacks specificity, making it challenging to drive the differential diagnosis. Ribs and scapulae are commonly affected by metastatic lesions (34, 35). Conversely, reports on brown tumors of the ribs are scarce (36, 37) and, to date, only two cases of brown tumor located in the scapula have been described (11, 14). Tsushima and colleagues reported a 38-year-old male patient with recurrent PC and multiple brown tumors involving the pelvis, bilateral ribs, right clavicle, left humerus and right scapula. Interestingly, in this case, bone lesions were not detected at the time of diagnosis but were identified during follow-up at functional imaging using 18-FDGPET/CT (18). Herein, we present the case of a 50 years-old male patient with PC and brown tumors affecting the rib and scapulae discovered at total-body CT scan.

Radiologically, brown tumors and bone metastases are poorly distinguishable on morphological imaging, such as CT scan. In fact, brown tumors typically present as irregular, single or multi-lobular, lytic lesion with or without sclerotic or cortical interruption (12, 37). These characteristics can overlap with those of bone metastases, making it difficult to differentiate between the two solely based on CT imaging. In our clinical case, the bony lesions were described as lytic lesions without sclerotic margins, raising suspicion for metastatic involvement. Additionally, it is worth noting that other functional imaging techniques may not reliably differentiate between brown tumors and metastatic lesions. Tc^99m^-Sestamibi scan, commonly used to localize hyperfunctioning parathyroid tissue, relies on the differential washout rate of the tracer from the parathyroid gland compared to the thyroid gland (38). Nowadays, parathyroid scintigraphy is commonly performed in conjunction with SPECT or SPECT/CT, where available, to provide a three-dimensional functional imaging of parathyroid tumors. When Tc^99m^ Sestamibi imaging visualizes bone lesions, there is a high suspicion for PC with metastatic involvement (39, 40). However, it is important to note that Tc^99m^ Sestamibi can be also taken up by brown tumors, mimicking metastatic lesions, as reported by several authors (41–44). The precise mechanisms underlying the increased uptake of sestamibi in a brown tumor remain unclear. However, it is likely due to increased perfusion, metabolism, and osteoclastic activity. Specifically, sestamibi uptake is associated with heightened cellular mitochondria content, enhanced blood flow, increased capillary permeability, and altered potassium diffusion potentials across mitochondrial and plasma membranes (38, 39).

Similarly, bone scintigraphy is unable to discriminate the nature of bone lesions. This imaging technique is highly sensitive and assesses the distribution of active bone formation induced by both benign and malignant lesions (45). The radiotracer used, ^99^mTc-labelled bisphosphonate, is taken up on the surface of hydroxyapatite based on the osteoblastic activity and the vascularization of the lesion (45). Indeed, elevated osteoblastic activity and increased vascularization can be observed in both brown tumors and metastatic lesions (8, 44, 46, 47). In fact, bony lesions in our patient exhibited uptake of both radiotracers used in parathyroid scintigraphy and bone scintigraphy. Furthermore, it is important to note that both brown tumors and bone metastases could be positive on positron emission tomography (PET)/CT with 2-[fluorine-18]-fluoro-2-deoxyD-glucose (^18^F-FDG) (11, 15, 29, 31). Unfortunately, this exam was not conducted in our patient. Therefore, in agreement with the literature as in our case, bone biopsy may be the only conclusive procedure for the differential diagnosis due to different histological characteristics of the two types of lesions (11, 13, 15, 37, 48). We would like to underline that we did not perform preoperative fine-needle aspiration of the parathyroid lesion, as this procedure is not recommended due to the risk of tumor rupture and seeding (14). Conversely, after discussion with the radiologist and counseling with the patient, due to the radiological characteristics and the particular site of the bone lesion, it was decided to perform a biopsy of the bone lytic lesion to differentiate between brown tumors and metastasis, as described in other case reports in the literature (11, 13, 15, 36, 47). Because of the risk of seeding tumor cells, we do not recommend bone biopsy for all patients, but only in selected cases. Patient characteristics should be individualized for treatment decisions.

Typically, metastases of PC share similarities to the primary tumor and may exhibit solid growth with tumor cells organized into cohesive masses of cells and/or trabecular growth. In addition to the presence of moderate atypia, fibrotic bands or necrotic areas may be present (49). Immunohistochemical analysis typically reveals positive staining for parathyroid hormone (PTH) and chromogranin A (50). Conversely, in cases like the one presented here, brown tumors are characterized by numerous giant cells with interstitial hemorrhage, hemosiderin deposits, microfracture, and growth of vascularized fibrous tissue rich in fibroblasts (3, 12).

The post-surgical follow-up confirms the benign nature of the brown tumor associated with PHPT which does not require specific treatments, other than the resolution of the underlying PHPT disease (51). Notably, in our patient partial re-ossification of the lesions was observed on CT scans and, as a result of the active new bone formation, they also showed greater uptake on bone scintigraphy performed after surgery. For some patients, surgical intervention may still be necessary in cases where fractures or large lesions cause compressive symptoms (30). However, in the presence of bone metastasis, particularly when solitary, surgery remains the primary therapeutic option to achieve sustained normalization of serum calcium levels (14).

Patients with severe PHPT and bone involvement are particularly susceptible to develop hungry bone syndrome. This condition arises from the significant transfer of calcium to the bone tissue following the removal of elevated circulating PTH levels after parathyroidectomy (52). Characteristic features include normal or elevated serum PTH, low serum phosphate and magnesium concentrations, and reduced 24-hour urinary calcium excretion. Risk factors for hungry bone syndrome include preoperative vitamin D deficiency, elevated preoperative PTH and alkaline phosphatase levels, large parathyroid tumors, and radiological evidence of bone disease. Acute hypocalcemia generally develops within 2 days post-surgery and may require intravenous calcium administration. This is typically followed by continuous infusion and oral calcium with activated vitamin D until the condition resolves (14).

## Conclusions

PC is a rare cause of PHPT with a poor prognosis, especially in the presence of distant metastases.

PC may manifest both metastatic lesions and brown tumors.

We show that while PHPT-related bone alterations are extremely rarer than in the past, they should still be taken into consideration in cases presenting with bone lesions, even in cases where malignancy is strongly suspected, in order to avoid unnecessary and potentially harmful surgical interventions.

## Data Availability

The datasets presented in this article are not readily available because of ethical and privacy restrictions. Requests to access the datasets should be directed to the corresponding author/s.
